# Optical Nonreciprocity in Asymmetric Optomechanical Couplers

**DOI:** 10.1038/srep08657

**Published:** 2015-03-02

**Authors:** Zheqi Wang, Lei Shi, Yi Liu, Xinbiao Xu, Xinliang Zhang

**Affiliations:** 1 Wuhan National Laboratory for Optoelectronics, Huazhong University of Science and Technology, Wuhan 430074, China

## Abstract

We propose an all-optical integrated nonreciprocal device on the optomechanical platform with a large nonreciprocal bandwidth and low operating power. The device is based on an asymmetric silicon coupler consisting of two branches. One of them is a conventional strip waveguide fixed on the substrate, and the other is a freestanding nanostring suspended above a groove in the substrate. When light is launched into the coupler, the optical gradient force between the freestanding nanostring and the underlying substrate leads to the deflection of the nanostring, and finally results in destruction of the initial phase-matching condition between the two branches. The suspended branch would achieve distinct deflections when light is incident from different ports. The simulation results show a nonreciprocal bandwidth of 13.1 nm with operating power of 390 μW. With the advantages of simple structure, low power consumption and large operating bandwidth, our work provides a promising solution for on-chip passive nonreciprocal device.

Silicon photonics is deemed as a promising candidate meeting the urgent requirements for ultra-low power consumption, ultra-high speed computing and ultra-high density communication. It provides a competitive complementary metal-oxide semiconductor (CMOS) compatible platform with ultra-compact scale and low fabricating cost. In the past decade, various components have been exploited on the silicon-on-insulator (SOI) platform, such as optical filter^1^, switch^2^, modulator^3^, isolator^4^ and polarization splitter^5^.

However, for the applications in nonlinear optics^6^, silicon seems unlike to completely fulfill this role since its Kerr nonlinear refractive index *n*_2_ is about only 6 × 10^−18^ m^2^·W^−1^. Considering that Kerr nonlinearity is an intrinsic material property, to achieve a certain refractive index variation Δ*n* = *n*_2_*I*, we often have to increase optical intensity *I*. High intensity would lead to some undesired results, such as high power consumption and additional insertion loss due to nonlinear absorption. To enhance the nonlinear effects in the silicon waveguide, schemes involved with thermo-optic effect^7^,^8^ and slow light effect^9^,^10^ are employed as alternative solutions. In addition, another extremely strong nonlinearity induced by optomechanical effect gets increasingly more attentions in recent years^11^,^12^.

In 2005, Povinelli et al. gives a theoretical investigation of the optical gradient force between parallel waveguides^13^. Since then, many optomechanical structures are proposed and demonstrated^14^,^15^,^16^,^17^,^18^, such as nanowaveguide^11^,^19^,^20^,^21^,^22^,^23^, microcavity^24^,^25^,^26^,^27^,^28^,^29^,^30^,^31^,^32^,^33^ and photonic crystal cavity^34^,^35^,^36^,^37^,^38^,^39^. The field of optomechanics has already been expanded to the single-photon level^40^. Various applications are also exploited, such as frequency comb^41^, Q-factor tunable microring resonator^42^, optical switch^43^ and optical detector^44^. The suspended waveguide deforms owing to optical gradient force and accordingly its effective index varies, which is called mechanical Kerr effect^11^,^12^,^45^. Mechanical Kerr effect could be up to 7 orders of magnitude larger than the conventional Kerr effect of materials^22^,^23^.

Among the photonic applications on SOI, optical nonreciprocal devices take a significant role. Optical nonreciprocity refers that light passes in one direction but gets blocked in the opposite direction. Generally, to realize optical nonreciprocity, Lorentz reciprocity must be broken^46^. Various methods are reported, such as those relying on magneto-optical effect^47^ and a time-dependent refractive index^48^,^49^,^50^,^51^. In addition, all-optical nonreciprocal approaches based on the Kerr nonlinearity and thermo-optic effect of materials have already been demonstrated^4^,^8^,^52^,^53^,^54^,^55^,^56^,^57^. Yet they are either with high operating power, e.g. 3.1 W^52^, or with a very narrow 10-dB nonreciprocal transmission bandwidth (NTB) and high insertion loss, e.g. 50 pm with 12 dB insertion loss^4^. There are also several schemes based on mechanical Kerr effect in optomechanical devices. In 2009, Manipatruni et al. demonstrated a delicate nonreciprocal device based on a Fabry-Perot cavity^58^. In 2012, Hafezi et al. proposed an intriguing theoretical scheme of nonreciprocal device consisting of a microtoroidal cavity optomechanical system^59^. Their bandwidths are 250 pm and a few MHz, respectively, which are still too narrow for practical applications.

In this paper, we propose a novel nonreciprocal structure based on an asymmetric optomechanical coupler. One of the coupler branches is a suspended nanostring, and the other is fixed on the substrate. When light passes through the suspended branch, the waveguide bends to the substrate and exhibits mechanical Kerr nonlinearity. Since the nanostring deflects at different levels according to routings of light, it manifests optical nonreciprocity. The device possesses a large 10-dB NTB of 13.1 nm with low input power of 390 μW.

## Results

### Principle of the optomechanical nonreciprocal device

**Figure 1:**
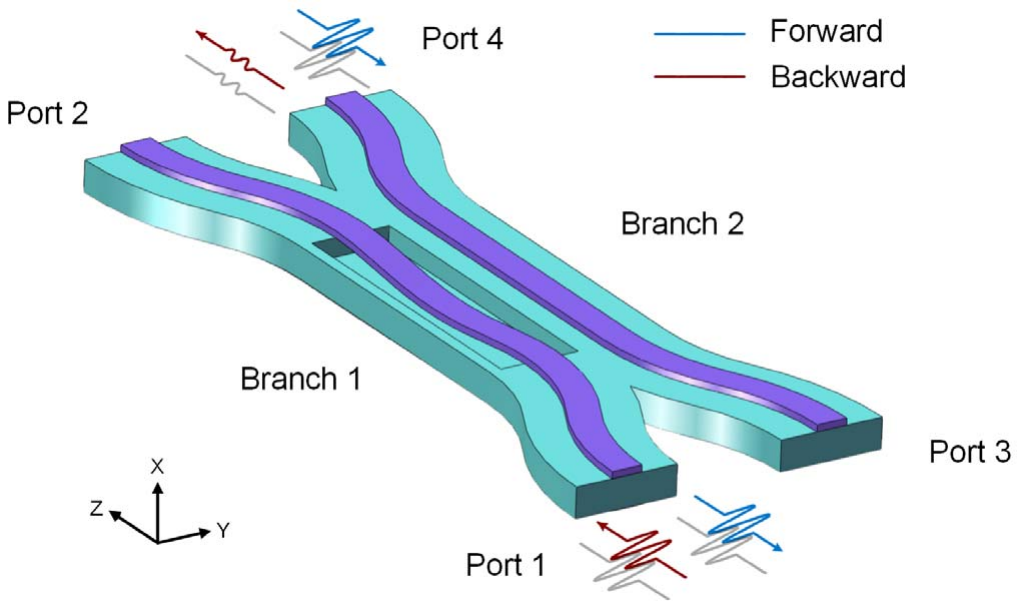
Schematic illustration of the asymmetric optomechanical coupler. Branch 1 is a freestanding silicon waveguide in close proximity to the underlying silicon dioxide substrate. Branch 2 is a conventional stripe waveguide fixed on the substrate. The backward transmission light is incident from Port 1, and emits from Port 4. The forward transmission route is just opposite.

Figure 1 shows the schematic illustration of the nonreciprocal device based on an asymmetric optomechanical coupler. The coupler comprises two branches. Branch 1 is a suspended double-clamped nanostring above a groove in the substrate. The groove is often obtained by etching the substrate with hydrofluoric acid. Branch 2 is fixed on the silicon dioxide substrate. When the incident power is very low, such as *P* = 1 μW, the device operates in the linear regime and the two branches satisfy the phase-matching condition. Thus the coupler shows reciprocal property. As a light beam exceeding the threshold power is launched, Branch 1 bends to the substrate and its effective index varies. Thereby the two branches no longer satisfy the initial phase-matching condition. Because the freestanding branch deforms at distinct levels according to the input ports, the device exhibits nonreciprocity. On the one hand, light incident from Port 4 gets transferred and outputs from Port 1 with very low insertion loss, and this is defined as the forward direction of light. On the other hand, light in the backward routing from Port 1 to Port 4 gets blocked.

### Device structure, mode distribution and optical gradient force

**Figure 2:**
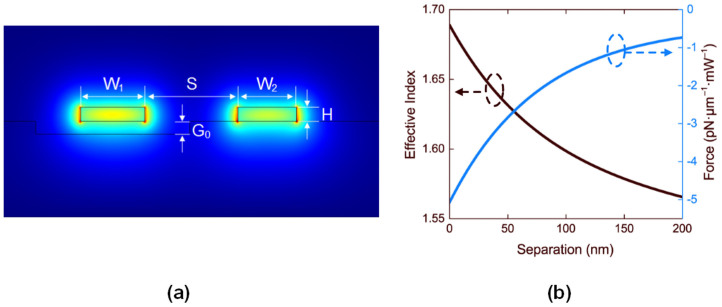
(a) Mode profile of the cross section. (b) Effective index and optical gradient force of Branch 1 versus the separation *d* between Branch 1 and the substrate.

As depicted in Figure 2(a), the cross section mode distribution at wavelength *λ*_0_ = 1550 nm is acquired with COMSOL Multiphysics, a commercial software based on finite element modeling method. Considering that Branch 1 is surrounded by air, it is slightly wider than Branch 2 to satisfy the phase-matching condition. The widths of the two branches are *W*_1_ = 500 nm and *W*_2_ = 455 nm, respectively. The heights of the branches are both *H* = 110 nm. The separation between the two waveguide *S* = 730 nm. The initial gap between the suspended waveguide and the substrate *G*_0_ = 100 nm. The device parameters have been demonstrated^60^. The length of the coupling region *L* is set to the half-beat length *L*_0_ = π/(2*κ*), where *κ* is the coupling coefficient. For a linear coupler, at the half-beat length *L*_0_ light incident from one branch would make a complete coupling to the other branch. Here in our coupler, the coupling coefficient *κ* = 1.468 × 10^4^ m^−1^, and the corresponding length of coupling region *L* = *L*_0_ = 107 μm.

As power ascends, the optical gradient force between Branch 1 and the underlying substrate causes that the suspended nanostring starts to deform. The optical gradient force of unit length and unit power *f* could be described as the follow^61^,^62^:
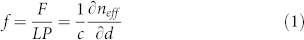
where *F* and *P* represent the force and the optical power in the whole waveguide, respectively. Here the variable *d* stands for the actual separation between the waveguide and the substrate when light is incident. In its initial non-deflection state, *d* = *G*_0_ = 100 nm. As indicated by Eq. (1), there is a positive correlation between the derivative of effective index and the optical force.

As shown in Figure 2(b), the effective index increases as the gap reduces. When the suspended branch gets closer to the substrate, in other word the separation *d* is smaller, there will be more proportion of optical field in the substrate and thus the effective index becomes higher. Furthermore, the effective index varies more rapidly when *d* is smaller. As a result, the corresponding optical gradient force gets larger when Branch 1 gets nearer to the substrate, where the minus sign represents an attractive force in the –X direction. As the interval between them becomes large enough, the force falls to zero. Here for the initial state, *d* = *G*_0_ = 100 nm, the optical gradient force *f* = −1.67 pN·μm^−1^·mW^−1^. Indeed there also exists a horizontal optical gradient force between the two coupler branches. Nonetheless as indicated by Eq. (1) the optical force between two media decays almost exponentially as their separation increases. Obviously the horizontal gap *S* is much larger than the vertical gap *G*_0_, 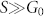. As a consequence, the horizontal optical force between Branch 1 and Branch 2 is far smaller than the vertical optical force between Branch 1 and the substrate, and thereby the effective index variation resulting from horizontal displacement is also far less than the vertical one. So for our device the horizontal gradient force could be neglected.

### Optical field distribution and the deflection along the Z direction

The light propagation properties are usually described with nonlinear coupler mode equations^63^:
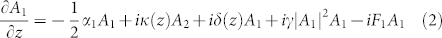

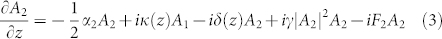
where *A* is the slowly varying complex amplitude, and *F* is the term of free carrier dispersion (FCD). The subscripts 1 and 2 denote Branch 1 and Branch 2, respectively. The loss *α* contains the linear loss *α*_Linear_, the loss caused by two photon absorption (TPA) *α*_TPA_, and the loss caused by free carrier absorption (FCA) *α*_FCA_. *Υ* and *δ* represent nonlinear coefficient and detuning, separately. The coupling coefficient *κ*(z) and detuning *δ*(z) vary as functions of Z due to the displacement distribution along the Z direction caused by the optical gradient force.

The deflection of the freestanding nanostring is determined by Euler Bernoulli beam theory:
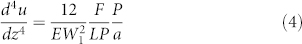
where *u*, usually a negative value, is the deflection of the nanostring. *E* = 131 × 10^9^ Pa is the Young's modulus of silicon, and *a* = *W*_1_ × *H* is the cross-section area of Branch 1. Here in our device, *d* = *G*_0_ + *u* = 100 [nm] + *u*. Substituting Eq. (1) into Eq. (4), we obtain the following equation:
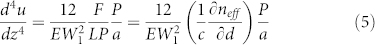
The double-clamped nanostring obeys the boundary conditions of *u*(0) = *u*′(0) = *u*(*L*) = *u*′(*L*) = 0.

### Optical switching characteristic and nonreciprocity

**Figure 3:**
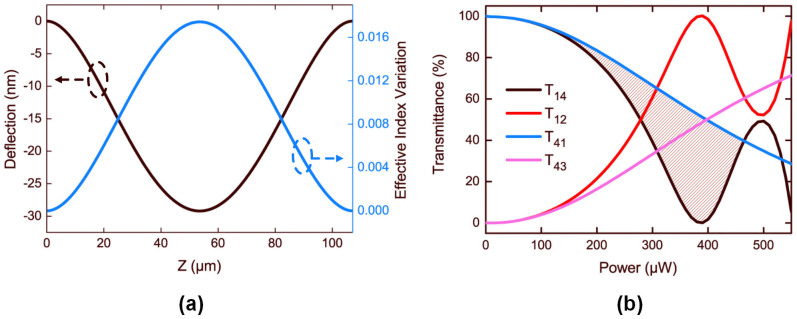
(a) Deflection of the freestanding Branch 1 and the corresponding effective index variation along the Z direction when the light is launched into Port 1. The negative sign of deflection represents that the branch deforms towards the substrate. (b) Transmittance (*T*_ij_) as a function of input power, where i and j are the numbers of input and output ports, respectively. Black and red solid curves: light is incident from the Port 1, and outputs from Port 4 of Branch 2 and Port 2 of Branch 1, respectively. Blue and magenta curve: light is incident from the Port 4, and then outputs from Port 1 of Branch 1 and Port 3 of Branch 2, respectively. *T*_14_ and *T*_41_ represent the backward and the forward transmission, separately.

The conventional Kerr nonlinear effect of materials is defined as *n* = *n*_0_ + *n*_2_*I*. Similarly, in the optomechanical device, the deflection of the suspended nanostring and the corresponding variation of effective index show positive correlation to the incident power. The mechanical Kerr effect could be evaluated by effective index changes at the point where the maximum displacement is achieved. *n*(*u* = *u*_max_) = *n*_0_ + Δ*n*_max_, where *u*_max_, usually a negative value, is the maximum deflection of the nanostring. The mechanical Kerr coefficient *Υ*_om_ and the mechanical Kerr index *n*_om_ are defined as the follow^12^,^45^: *Υ*_om_ = *k*_0_Δ*n*_max_/*P*, *n*_om_ = *A*_eff_Δ*n*_max_/*P*, where *k*_0_ is the wavenumber in vacuum and *A*_eff_ is the effective area. As Figure 3(a) depicts, at the point where the maximum deflection is achieved, the effective index variation is the largest. Here the incident power *P* = 390 μW, the maximum deflection |*u*_max_| = 29 nm, and its corresponding effective index variation is 0.0174. Thus it could be derived that *n*_om_ = 6.1 × 10^−12^ m^2^·W^−1^ and *Υ*_om_ = 1.8 × 10^8^ m^−1^·W^−1^.

As the incident power continues to rise, the device doesn't remain in the linear regime, and shows nonlinear switching characteristic and nonreciprocity. As mentioned, the detuning *δ* between the two branches due to the nanostring deflection is power dependent, and therefore the transmittance of the device is also power dependent. Taking the black curve *T*_14_ in Fig. 3(b) for instance, when the light is incident from Port 1, the transmittance at Port 4 falls as power rises, from 100% at *P* ~ 1 μW down to approximate to zero at *P* = 390 μW. Then as the power increases continuously, some properties such as the period of coupling changes and the zero-output condition is not satisfied any more. Just like the nonlinear optical switching based on conventional Kerr nonlinear effect, the transmittance bounces off zero as power continues to rise.

Likewise, when light is incident from Port 4 on the fixed branch, there is also a power dependent switching characteristic. However, because the power proportions in the suspended waveguide are different, the deflections of the suspended branch and the effective index variations achieved are distinct. The maximum deflections of the freestanding nanostring are 15 nm and 29 nm for the forward and the backward transmission, respectively. The distinction finally leads to the difference in their switching thresholds, ~400 μW for the forward transmission and ~280 μW for the opposite direction. As indicated in Fig. 3(b), the black curve of the backward transmission doesn't overlap the blue curve of the forward transmission, in other word *T*_14_ ≠ *T*_41_. The shadow region in Fig. 3(b), manifests a nonreciprocal transmission window.

**Figure 4:**
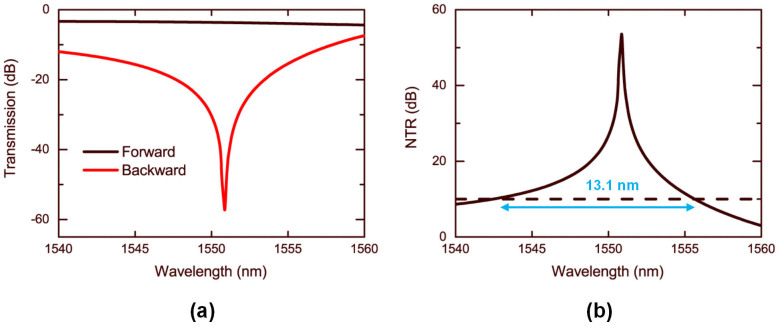
(a) The forward and the backward transmission spectra, with incident power *P* = 390 μW. (b) The NTR spectrum.

As portrayed in Figure 4, with incident power of 390 μW, the transmission spectra of the forward and the backward are apparently different. At the wavelength of 1550.85 nm, the forward transmission is −3.7 dB and the backward transmission is only −58.2 dB. Thus the nonreciprocal transmission ratio (NTR) reaches its peak of 54.5 dB. Since resonance structures are not employed in our scheme, there is no inherent bandwidth limit and the bandwidth is relatively large. The 10-dB NTB is 13.1 nm corresponding to a wavelength range from 1542.5 nm to 1555.6 nm. In addition, there are also no nonlinear losses owing to low operating power. As a consequence, the insertion loss is less than 3.9 dB in the nonreciprocal band.

## Discussion

**Figure 5:**
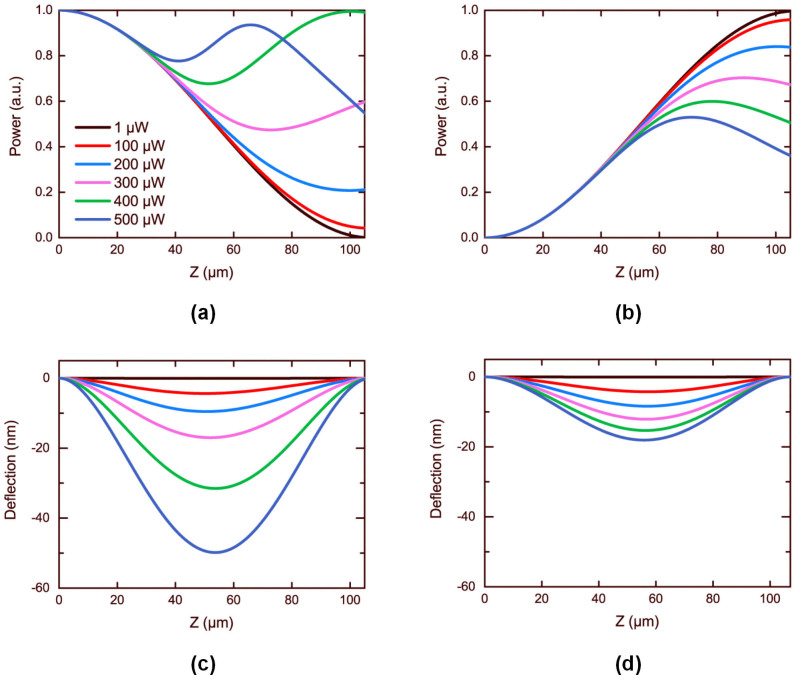
(a) and (b) Normalized power distribution in Branch 1 along the Z direction. (c) and (d) Deflection of the freestanding nanostring along the Z direction. (a) and (c) Light is launched into Port 1. (b) and (d) Light is launched into Port 4. The black curves show the linear properties of the coupler, at very low power *P* = 1 μW. The rest curves show the situations corresponding to larger powers from 100 μW to 500 μW.

Figures 5(a) and 5(b) show the power distribution along the Z direction for light incident from Port 1 and Port 4, respectively. Figs. 5(c) and 5(d) display the corresponding deflections of the nanostring, respectively. As indicated by the black curves in Figs. 5(c) and 5(d), ultra-low incident power such as *P* = 1 μW is insufficient to deform the nanostring. Thus the device functions in the linear regime and light gets transferred to the other branch as depicted by the black curves in Figs. 5(a) and 5(b). As shown in Figs. 5(c) and 5(d), when the power ascends the nanostring bends more heavily to the substrate and the coupler doesn't remain in the linear regime.

In the conventional case, there is only one individual independent suspended nanostring, and thus light power is uniform along the waveguide. The deflection of the nanostring is often described with the analytic expression as the follow^11^:
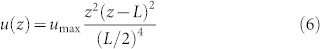
where *u*_max_ is always achieved at the center of the freestanding nanostring *z* = *L*/2. Here in our coupler, the deflection of one branch approximately accords with the expression, but *u*_max_ is different. *u*_max_ depends on not only power in the nanostring but also the power distribution. As illustrated in Figs. 5(a) and 5(b), it is very obvious that more power is in Branch 1 for the backward transmission than the opposite. As a result, the deflection in Fig. 5(c) is larger than that in Fig. 5(d) with the same incident power. On the other hand, the different power distribution would cause different deflections. In our device the power is non-uniform along the Z direction. According to Equations (4) and (5), the same force acts on the center of the nanostring leads to a larger deflection than on the point off the center.

Thermal noises would lead to deflection of the suspended nanostring even without incident light. The thermal noise arising from thermal Brownian motion is characterized by the root-mean-square displacement amplitude *u*_rms_^64^,^65^,^66^,^67^,
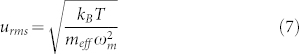
where *ω*_m_ is the fundamental mechanical frequency, *m*_eff_ is the effective mass of the beam, *k*_B_ is Boltzmann constant, and *T* is the ambient temperature (300 K). The natural frequency of the *n*th mechanical mode *ω^n^_m_* is described by the equation^62^,
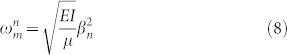
where *μ* is the mass per unit length, and *I* is the second moment of area. For a double-clamped beam, *β*_n_ satisfies the equation cos(*β*_n_*L*) cosh(*β*_n_*L*) − 1 = 0. For the fundamental mechanical mode, *β*_1_*L* = 4.73004. Here the fundamental mechanical frequency of our device *ω*_m_ = 4.64 × 10^5^ rad/s, and the root-mean-square displacement amplitude *u*_rms_ = 1.18 nm. Since *ω*_m_ ∝ *H*/*L*^2^, to increase natural mechanical frequencies, we could reduce *L* and increase *H*. Since *u*_rms_ ∝ *T*^1/2^*L*^3/2^/*H*^3/2^*W*_1_^1/2^, in order to suppress thermal noises, we could reduce *L*, and increase *H* and *W*_1_. In addition as the ambient temperature *T* falls, thermal noises would be also lower accordingly.

## Conclusion

In summary, we proposed an asymmetric optomechanical coupler exhibiting nonreciprocity induced by mechanical Kerr effect. The device shows a large NTB, high NTR, low insertion loss, and low operating power. This work provides a promising solution for all-optical nonreciprocal device on a silicon chip.

## Methods

The nonlinear coupled mode equations are solved by a differential method. In the equations, the parameters *κ* and *δ* are given as the follow^68^,
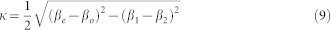

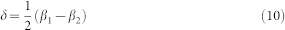
where *β*_e_ and *β*_o_ are the propagation constants of even supermode and odd supermode, respectively; *β*_1_ and *β*_2_ are the propagation constants of individual guided modes in Branch 1 and Branch 2, respectively. The propagation constants of the cross section are calculated by 2D mode analysis of COMSOL Multiphysics.

Under continuous wave conditions, the final solution is a stationary solution. The coupled mode Eqs. (2) and (3), and the mechanical Eqs. (4) and (5), are jointly solved with self-made MATLAB codes by an iteration method, until the difference between two calculation results is less than the tolerant error.

**Figure 6:**
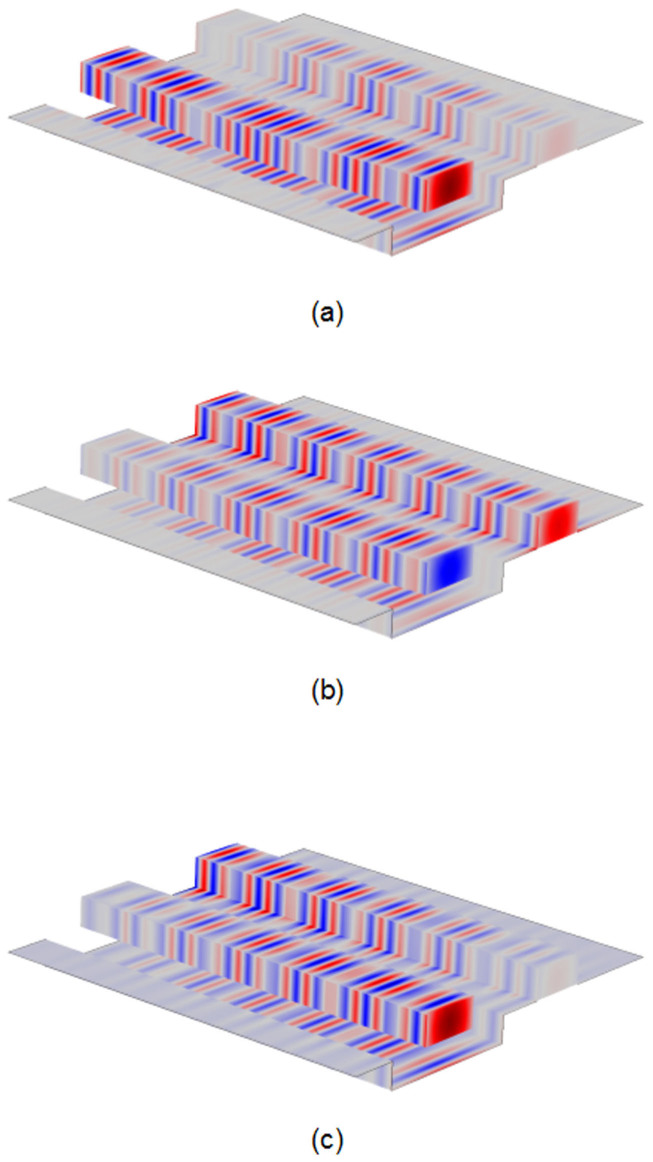
Y component of electric field along the waveguide. (a) Light is launched into Port 1, *P* = 390 μW. (b) Light is launched into Port 4, *P* = 390 μW. (c) Light is incident from Port 1 and outputs from Port 4, *P* = 1 μW.

Then the optical field distribution of the coupler with the calculated waveguide deformation is also verified by 3D simulations in COMSOL wave optics module. As shown in Fig. 6, the simulation results fit well with the results given by the self-made MATLAB code.

## Author Contributions

Z.Q.W. and L.S. conceived the design. Z.Q.W. performed the numerical simulations, and Y.L. assisted with the numerical simulation. Z.Q.W. analyzed the data, and X.B.X. assisted with the data analysis. Z.Q.W. prepared the figures and wrote the manuscript. L.S. and X.L.Z. edited the manuscript. L.S. supervised the project. All authors discussed the results and commented on the manuscript.

## References

[b1] Xia F., Rooks M., Sekaric L. & Vlasov Y. Ultra-compact high order ring resonator filters using submicron silicon photonic wires for on-chip optical interconnects. Opt. Express 15, 11934–11941 (2007). 19547556 10.1364/oe.15.011934

[b2] Almeida V. R., Barrios C. A., Panepucci R. R. & Lipson M. All-optical control of light on a silicon chip. Nature 431, 1081–1084 (2004). 15510144 10.1038/nature02921

[b3] Reed G. T., Mashanovich G., Gardes F. Y. & Thomson D. J. Silicon optical modulators. Nat. Photonics 4, 518–526 (2010).

[b4] Fan L. *et al.* An All-Silicon Passive Optical Diode. Science 335, 447–450 (2012). 22194410 10.1126/science.1214383 PMC5563475

[b5] Fukuda H. *et al.* Ultrasmall polarization splitter based on silicon wire waveguides. Opt. Express 14, 12401–12408 (2006). 19529672 10.1364/oe.14.012401

[b6] Leuthold J., Koos C. & Freude W. Nonlinear silicon photonics. Nat. Photonics 4, 535–544 (2010).

[b7] Xu Q. & Lipson M. Carrier-induced optical bistability in silicon ring resonators. Opt. Lett. 31, 341–343 (2006). 16480202 10.1364/ol.31.000341

[b8] Wang J. *et al.* A Theoretical Model for an Optical Diode Built With Nonlinear Silicon Microrings. J. Lightwave Technol. 31, 313–321 (2013).

[b9] Baba T. Slow light in photonic crystals. Nat. Photonics 2, 465–473 (2008).

[b10] Boyd R. W. & Gauthier D. J. Controlling the Velocity of Light Pulses. Science 326, 1074–1077 (2009). 19965419 10.1126/science.1170885

[b11] Pernice W. H. P., Li M. & Tang H. X. A mechanical Kerr effect in deformable photonic media. Appl. Phys. Lett. 95, 123507 (2009).

[b12] Ma J. & Povinelli M. L. Mechanical Kerr nonlinearities due to bipolar optical forces between deformable silicon waveguides. Opt. Express 19, 10102–10110 (2011). 21643268 10.1364/OE.19.010102

[b13] Povinelli M. L. *et al.* Evanescent-wave bonding between optical waveguides. Opt. Lett. 30, 3042–3044 (2005). 16315715 10.1364/ol.30.003042

[b14] Kippenberg T. J. & Vahala K. J. Cavity Opto-Mechanics. Opt. Express 15, 17172–17205 (2007). 19551012 10.1364/oe.15.017172

[b15] Favero I. & Karrai K. Optomechanics of deformable optical cavities. Nat. Photonics 3, 201–205 (2009).

[b16] Van Thourhout D. & Roels J. Optomechanical device actuation through the optical gradient force. Nat. Photonics 4, 211–217 (2010).

[b17] Ma J. & Povinelli M. L. Applications of optomechanical effects for on-chip manipulation of light signals. Curr. Opin. Solid State Mater. Sci. 16, 82–90 (2012).

[b18] Metcalfe M. Applications of cavity optomechanics. Appl. Phys. Rev. 1, 031105 (2014).

[b19] Pernice W. H. P., Li M. & Tang H. X. Theoretical investigation of the transverse optical force between a silicon nanowire waveguide and a substrate. Opt. Express 17, 1806–1816 (2009). 19189011 10.1364/oe.17.001806

[b20] Li M., Pernice W. H. P. & Tang H. X. Tunable bipolar optical interactions between guided lightwaves. Nat. Photonics 3, 464–468 (2009).

[b21] Roels J. *et al.* Tunable optical forces between nanophotonic waveguides. Nat. Nanotechnol. 4, 510–513 (2009). 19662013 10.1038/nnano.2009.186

[b22] Butsch A., Conti C., Biancalana F. & Russell P. S. J. Optomechanical Self-Channeling of Light in a Suspended Planar Dual-Nanoweb Waveguide. Phys. Rev. Lett. 108, 093903 (2012). 22463639 10.1103/PhysRevLett.108.093903

[b23] Conti C., Butsch A., Biancalana F. & Russell P. S. J. Dynamics of optomechanical spatial solitons in dual-nanoweb structures. Phys. Rev. A. 86, 013830 (2012). 10.1103/PhysRevLett.108.093903 22463639

[b24] Rakich P. T., Popovic M. A., Soljacic M. & Ippen E. P. Trapping, corralling and spectral bonding of optical resonances through optically induced potentials. Nat. Photonics 1, 658–665 (2007).

[b25] Wiederhecker G. S., Chen L., Gondarenko A. & Lipson M. Controlling photonic structures using optical forces. Nature 462, 633–636 (2009). 19915549 10.1038/nature08584

[b26] Rosenberg J., Lin Q. & Painter O. Static and dynamic wavelength routing via the gradient optical force. Nat. Photonics 3, 478–483 (2009).

[b27] Povinelli M. *et al.* High-Q enhancement of attractive and repulsive optical forces between coupled whispering-gallery- mode resonators. Opt. Express 13, 8286–8295 (2005). 19498858 10.1364/opex.13.008286

[b28] Carmon T. & Vahala K. J. Modal Spectroscopy of Optoexcited Vibrations of a Micron-Scale On-Chip Resonator at Greater than 1 GHz Frequency. Phys. Rev. Lett. 98, 123901 (2007). 17501123 10.1103/PhysRevLett.98.123901

[b29] Anetsberger G., Riviere R., Schliesser A., Arcizet O. & Kippenberg T. J. Ultralow-dissipation optomechanical resonators on a chip. Nat. Photonics 2, 627–633 (2008).

[b30] Anetsberger G. *et al.* Near-field cavity optomechanics with nanomechanical oscillators. Nat. Phys. 5, 909–914 (2009).

[b31] Weis S. *et al.* Optomechanically Induced Transparency. Science 330, 1520–1523 (2010). 21071628 10.1126/science.1195596

[b32] Verhagen E., Deleglise S., Weis S., Schliesser A. & Kippenberg T. J. Quantum-coherent coupling of a mechanical oscillator to an optical cavity mode. Nature 482, 63–67 (2012). 22297970 10.1038/nature10787

[b33] Eichenfield M., Michael C. P., Perahia R. & Painter O. Actuation of micro-optomechanical systems via cavity-enhanced optical dipole forces. Nat. Photonics 1, 416–422 (2007).

[b34] Eichenfield M., Camacho R., Chan J., Vahala K. J. & Painter O. A picogram- and nanometre-scale photonic-crystal optomechanical cavity. Nature 459, 550–555 (2009). 19489118 10.1038/nature08061

[b35] Eichenfield M., Chan J., Camacho R. M., Vahala K. J. & Painter O. Optomechanical crystals. Nature 462, 78–82 (2009). 19838165 10.1038/nature08524

[b36] Alegre T. P. M., Perahia R. & Painter O. Optomechanical zipper cavity lasers: theoretical analysis of tuning range and stability. Opt. Express 18, 7872–7885 (2010). 20588628 10.1364/OE.18.007872

[b37] Safavi-Naeini A. H. *et al.* Electromagnetically induced transparency and slow light with optomechanics. Nature 472, 69–73 (2011). 21412237 10.1038/nature09933

[b38] Chan J. *et al.* Laser cooling of a nanomechanical oscillator into its quantum ground state. Nature 478, 89–92 (2011). 21979049 10.1038/nature10461

[b39] Notomi M., Taniyama H., Mitsugi S. & Kuramochi E. Optomechanical Wavelength and Energy Conversion in High-Q Double-Layer Cavities of Photonic Crystal Slabs. Phys. Rev. Lett. 97, 023903 (2006). 16907448 10.1103/PhysRevLett.97.023903

[b40] Nunnenkamp A., Borkje K. & Girvin S. M. Single-Photon Optomechanics. Phys. Rev. Lett. 107 (2011). 10.1103/PhysRevLett.107.063602 21902323

[b41] Butsch A., Koehler J. R., Noskov R. E. & Russell P. S. J. CW-pumped single-pass frequency comb generation by resonant optomechanical nonlinearity in dual-nanoweb fiber. Optica. 1, 158–164 (2014).

[b42] Long Y. & Wang J. Optically-controlled extinction ratio and Q-factor tunable silicon microring resonators based on optical forces. Sci. Rep. 4, 5409; 10.1038/srep05409 (2014). 24958225 PMC4067623

[b43] Fong K. Y., Pernice W. H. P., Li M. & Tang H. X. Tunable optical coupler controlled by optical gradient forces. Opt. Express 19, 15098–15108 (2011). 21934871 10.1364/OE.19.015098

[b44] Bagci T. *et al.* Optical detection of radio waves through a nanomechanical transducer. Nature 507, 81–85 (2014). 24598636 10.1038/nature13029

[b45] Yu Y. F. *et al.* Force-induced optical nonlinearity and Kerr-like coefficient in opto-mechanical ring resonators. Opt. Express 20, 18005–18015 (2012). 23038348 10.1364/OE.20.018005

[b46] Jalas D. *et al.* What is - and what is not - an optical isolator. Nat. Photonics 7, 579–582 (2013).

[b47] Bi L. *et al.* On-chip optical isolation in monolithically integrated non-reciprocal optical resonators. Nat. Photonics 5, 758–762 (2011).

[b48] Yu Z. & Fan S. Complete optical isolation created by indirect interband photonic transitions. Nat. Photonics 3, 91–94 (2009).

[b49] Kang M. S., Butsch A. & Russell P. S. J. Reconfigurable light-driven opto-acoustic isolators in photonic crystal fibre. Nat. Photonics 5, 549–553 (2011).

[b50] Lira H., Yu Z., Fan S. & Lipson M. Electrically driven nonreciprocity induced by interband photonic transition on a silicon chip. Phys. Rev. Lett. 109, 033901 (2012). 22861851 10.1103/PhysRevLett.109.033901

[b51] Tzuang L. D., Fang K., Nussenzveig P., Fan S. & Lipson M. Non-reciprocal phase shift induced by an effective magnetic flux for light. Nat. Photonics 8, 701–705 (2014).

[b52] Gallo K., Assanto G., Parameswaran K. R. & Fejer M. M. All-optical diode in a periodically poled lithium niobate waveguide. Appl. Phys. Lett. 79, 314–316 (2001).

[b53] Soljacic M., Luo C., Joannopoulos J. D. & Fan S. Nonlinear photonic crystal microdevices for optical integration. Opt. Lett. 28, 637–639 (2003). 12703925 10.1364/ol.28.000637

[b54] Poulton C. G. *et al.* Design for broadband on-chip isolator using stimulated Brillouin scattering in dispersion-engineered chalcogenide waveguides. Opt. Express 20, 21235–21246 (2012). 23037247 10.1364/OE.20.021235

[b55] Zhang Y. *et al.* Silicon optical diode based on cascaded photonic crystal cavities. Opt. Lett. 39, 1370–1373 (2014). 24690790 10.1364/OL.39.001370

[b56] Xu M. *et al.* Push-Pull Optical Nonreciprocal Transmission in Cascaded Silicon Microring Resonators. IEEE Photonics J. 5, 2200307–2200307 (2013).

[b57] Fan L. *et al.* Silicon optical diode with 40 dB nonreciprocal transmission. Opt. Lett. 38, 1259–1261 (2013). 23595451 10.1364/OL.38.001259

[b58] Manipatruni S., Robinson J. T. & Lipson M. Optical Nonreciprocity in Optomechanical Structures. Phys. Rev. Lett. 102, 213903 (2009). 19519108 10.1103/PhysRevLett.102.213903

[b59] Hafezi M. & Rabl P. Optomechanically induced non-reciprocity in microring resonators. Opt. Express 20, 7672–7684 (2012). 22453446 10.1364/OE.20.007672

[b60] Li M. *et al.* Harnessing optical forces in integrated photonic circuits. Nature 456, 480–484 (2008). 19037311 10.1038/nature07545

[b61] Rakich P. T., Popovic M. A. & Wang Z. General treatment of optical forces and potentials in mechanically variable photonic systems. Opt. Express 17, 18116–18135 (2009). 19907602 10.1364/OE.17.018116

[b62] Guo X., Zou C.-L., Ren X.-F., Sun F.-W. & Guo G.-C. Broadband opto-mechanical phase shifter for photonic integrated circuits. Appl. Phys. Lett. 101, 071114 (2012).

[b63] Agrawal G. P. Applications of nonlinear fiber optics. (Academic Press, 2008).

[b64] Sun X., Zheng J., Poot M., Wong C. W. & Tang H. X. Femtogram Doubly Clamped Nanomechanical Resonators Embedded in a High-Q Two-Dimensional Photonic Crystal Nanocavity. Nano Lett. 12, 2299–2305 (2012). 22471420 10.1021/nl300142t

[b65] Basarir O., Bramhavar S. & Ekinci K. L. Near-field optical transducer for nanomechanical resonators. Appl. Phys. Lett. 97, 253114 (2010).

[b66] Basarir O., Bramhavar S. & Ekinci K. L. Monolithic integration of a nanomechanical resonator to an optical microdisk cavity. Opt. Express 20, 4272–4279 (2012). 22418186 10.1364/OE.20.004272

[b67] Hu Y.-W., Xiao Y.-F., Liu Y.-C. & Gong Q. Optomechanical sensing with on-chip microcavities. Front. Phys. 8, 475–490 (2013).

[b68] Okamoto K. Fundamentals of Optical Waveguides. (Academic Press, 2010).

